# Strategies of Dose Escalation in the Treatment of Locally Advanced Non-Small Cell Lung Cancer: Image Guidance and Beyond

**DOI:** 10.3389/fonc.2014.00156

**Published:** 2014-06-20

**Authors:** Alexander Chi, Nam Phong Nguyen, James S. Welsh, William Tse, Manish Monga, Olusola Oduntan, Mohammed Almubarak, John Rogers, Scot C. Remick, David Gius

**Affiliations:** ^1^Department of Radiation Oncology, Mary Babb Randolph Cancer Center of West Virginia University, Morgantown, WV, USA; ^2^The International Geriatric Radiotherapy Group, Tucson, AZ, USA; ^3^Northern Illinois University Institute for Neutron Therapy at Fermilab, Batavia, IL, USA; ^4^Division of Hematology and Oncology, Mary Babb Randolph Cancer Center of West Virginia University, Morgantown, WV, USA; ^5^Thoracic Surgery, Mary Babb Randolph Cancer Center of West Virginia University, Morgantown, WV, USA; ^6^Department of Radiation Oncology, Robert H. Lurie Comprehensive Cancer Center of Northwestern University, Chicago, IL, USA

**Keywords:** image guidance, intensity-modulated radiotherapy, NSCLC, adaptive radiotherapy, proton therapy

## Abstract

Radiation dose in the setting of chemo-radiation for locally advanced non-small cell lung cancer (NSCLC) has been historically limited by the risk of normal tissue toxicity and this has been hypothesized to correlate with the poor results in regard to local tumor recurrences. Dose escalation, as a means to improve local control, with concurrent chemotherapy has been shown to be feasible with three-dimensional conformal radiotherapy in early phase studies with good clinical outcome. However, the potential superiority of moderate dose escalation to 74 Gy has not been shown in phase III randomized studies. In this review, the limitations in target volume definition in previous studies; and the factors that may be critical to safe dose escalation in the treatment of locally advanced NSCLC, such as respiratory motion management, image guidance, intensity modulation, FDG–positron emission tomography incorporation in the treatment planning process, and adaptive radiotherapy, are discussed. These factors, along with novel treatment approaches that have emerged in recent years, are proposed to warrant further investigation in future trials in a more comprehensive and integrated fashion.

## Introduction

Concurrent chemo-radiation is the standard of care in the management of non-small cell lung cancer (NSCLC) after its superiority over radiotherapy alone or sequential chemo-radiation has been demonstrated in multiple phase III randomized trials (1–5). In a meta-analysis of 1205 patients with locally advanced NSCLC from six randomized studies, concurrent chemo-radiation decreased loco-regional progression by 6.1% at 5 years when compared with sequential chemo-radiation (28.9 vs. 35.0%, *p* = 0.01) (6). This resulted in an improvement in overall survival of 4.5% at 5 years (15.1 vs. 10.6%, *p* = 0.004) and as suggested by the authors of this study, survival may be directly related to loco-regional control. The risk of regional failure, i.e., in the mediastinum, is relatively low after chemo-radiation to a dose of approximately 60 Gy with conventional fractionation (5, 7); in contrast, the rate of local failure remains relatively high. As such, it seems reasonable to propose that techniques to improve primary tumor control through dose escalation may be one strategy to improve treatment outcome in locally advanced NSCLC.

Dose escalation in the treatment of stage I–III NSCLC has been shown to be feasible in multiple institutional and early phase prospective studies (8–11). Among them, the radiation dose has been found to be a significant predictor of local control and survival by many (8–10). Based on the clinical outcome from a University of Michigan study, 84.5 Gy was found to be required to achieve a local progression free survival (LPFS) of 50% at 30 months in patients with NSCLC (12). Further radiobiological modeling has suggested that a biologically effective dose (BED) of over 100 Gy_10_ is required to achieve a LPFS of ≥80% at 30 months in the treatment of NSCLC (13). This has been validated clinically in the treatment of early stage NSCLC with stereotactic ablative radiotherapy (SABR), which is also referred to as stereotactic body radiation therapy (SBRT) (14–16). In locally advanced NSCLC, every 1 Gy_10_ in dose escalation was found to be associated with a 4% increase in survival and a 3% increase *in loco*-regional control in past chemo-radiation trials conducted by the Radiation Therapy Oncology Group (RTOG) (17). When combined with weekly carboplatin and paclitaxel, a maximally tolerated dose (MTD) of 74 Gy delivered with three-dimensional (3D) technique was found in a phase I/II RTOG study, RTOG 0117 (18). Inoperable patients with stage I–III NSCLC were included in this study, and a median survival of 21.6 months was observed in stage III patients. In a similar phase II study, a median survival of 24.3 months in stage III NSCLC patients was observed after induction carboplatin/paclitaxel followed by concurrent carboplatin/paclitaxel and radiation to 74 Gy (19).

Based on early clinical evidence, a phase III randomized dose escalation trial (RTOG 0617) was conducted by the RTOG. In this study, patients were randomized to chemo-radiation to 60 vs. 74 Gy, and with or without Cetuximab (four arms) (20). Despite the anticipated improvement in clinical outcome with dose escalation, it actually resulted in inferior median survival (19.5 vs. 28.7 months, *p* = 0.0007) and an increase in local failure at 18 months (34.3 vs. 25.1%, *p* = 0.0319). In addition, dose escalation also resulted in an increase in grade 3 esophagitis (20.9 vs. 7.0%, *p* = 0.0003). The causes of poorer outcome in the 74-Gy arms remain to be discerned. However, several factors may potentially contribute to this finding, such as, larger-than-necessary planning target volume (PTV) in patients receiving 3D conformal radiotherapy (3D-CRT), which potentially leads to increased normal tissue toxicity; suboptimal target volume delineation when 18F-fluorodeoxyglucose (FDG) positron emission tomography (PET) information is not incorporated in the treatment planning process; failure to account for tumor shrinkage during the course of radiotherapy; and delayed tumor cell repopulation associated with prolonged overall treatment time. These can potentially be minimized with image-guided, intensity-modulated radiotherapy (IG-IMRT), and adaptive radiotherapy (ART), which may improve the efficacy of dose escalation in the treatment of locally advanced NSCLC in future clinical trials.

## Target Volume in Relation to Clinical Outcome and Toxicities

In the treatment of locally advanced NSCLC with chemo-radiation, large tumor margins are often used to account for respiratory motion and set up uncertainties in the era of conventional and 3D radiotherapy (Table 1). With the addition of elective nodal irradiation (ENI), an even larger volume of normal tissue is included within the treated volume. As shown in Table 1, multiple clinical trials investigating the efficacy of sequential and concurrent chemo-radiation frequently used margins of 1.5–2 cm for the primary tumor. ENI was often carried out in these studies with inclusion of the bilateral hilum, mediastinum, and the ipsilateral supra-clavicular fossa in the initial radiation field, which extended 4–5 cm below the carina (3–5, 21–23). In these studies, dose to the gross disease has been limited to approximately 60 Gy with poor clinical outcome and fatal treatment related toxicities reported. Concurrent chemo-radiation prior to RTOG 0617 often led to a local control of 60–83%, while the median survival with concurrent chemo-radiation often increased to >15 months (3–5). Among them, seven late fatal pulmonary toxicities were observed in RTOG 9410, which demonstrated no significant improvement in local control and only marginal improvement in median survival with concurrent chemo-radiation over sequential chemo-radiation (5). These findings suggest that the large tumor volumes treated in the past not only precludes dose escalation to the primary tumor, but also increase the risk of severe treatment related toxicity.

**Table 1:** **Target volume, toxicity, and clinical outcome in selected phase III randomized trials and phase I/II dose escalation trials combining chemotherapy and radiation for locally advanced NSCLC**.

References	Radiotherapy technique	Tumor margin/target volume expansion	ENI	Radiation dose	Severe RT related toxicity	Local control	Median survival (months)
**PHASE III**							
Dillman et al. (21)	2D	1.5 cm	Yes	60 Gy	1% (esophagitis, pneumonitis) in each arm	OR: ChemoRT 56% RT 43% (*p* = 0.092)	ChemoRT 13.7 (*p* = 0.034) RT 9.6
Sause et al. (22)	2D	2 cm	Yes	60, 69.6 Gy (bid)	Two radiation related deaths on ChemoRT and hyperfrx arms (1 on each arm)		RT 11.4 ChemoRT 13.2 (*p* = 0.04) Hyperfrx RT 12
Le Chevalier et al. (23)	2D	1–1.5 cm	Yes	65 Gy	Eight treatment related fatal toxicities	At 1 year: RT 17% ChemoRT 15%	RT 10 ChemoRT 12 (*p* = 0.02)
Furuse et al. (3)	2D	1.5 cm	Yes	56 Gy	No >grade 3 esophagitis, 1 grade 4 pulmonary toxicity on each arm	OR: concurrent 84.0% Sequential 66.4% (*p* = 0.0002)	Concurrent 16.5 (*p* = 0.03998) Sequential 13.3
Zatloukal et al. (4)	3D	1.5–2 cm	Yes	60 Gy	Severe acute esophagitis (*p* = 0.009): sequential 4% Concurrent 18%	Local only (*p* = NS): sequential 42% Concurrent 60%	Sequential 12.9 Concurrent 16.6
Curran et al. (5)	2D	2–2.5 cm	Yes	63; 69.6 Gy (bid)	Acute esophagitis (*p* < 0.001): sequential 4% Concurrent 22% Concurrent hyperfrx 45% Late severe pulmonary toxicities: no difference between three arms (13–17%) Seven late fatal pulmonary toxicities observed in this study	Sequential 61% Concurrent 70% Concurrent hyperfrx 71% (*p* = NS)	Sequential 14.6 Concurrent 17.0 Concurrent hyperfrx 15.6
Nyman et al. (7)	3D	CTV: 1.5–2 cm	No	64.6 Gy (bid); 60 Gy	Severe esophagitis: concurrent hyperfrx 20% Concurrent, daily 8% Concurrent, weekly 19% Severe pneumonitis: concurrent hyperfrx 0 Concurrent, daily 3% Concurrent, weekly 3%	Concurrent Hyperfrx 78% Concurrent, daily 78% Concurrent, weekly 83%	Concurrent Hyperfrx 17.69 Concurrent, daily 17.68 Concurrent, weekly 20.63
Bradley et al. (20)	3D/IMRT	CTV: 0.5–1 cm; ITV (no 4D): 0.5–1 cm PTV: 0.5–1 cm	No	60 Gy; 74 Gy	Increased grade 5 toxicity observed with 74 Gy	18-month local failure: 60 Gy 25.1% 74 Gy 34.3% (*p* = 0.0319)	60 Gy 28.7 74 Gy 19.5
**PHASE I, II**							
Bradley et al. (18)	3D	GTV–PTV: 1–1.5 cm	No	74 Gy	Grade 5 acute toxicity 4% Late grade 3–4 toxicities 20%		21.6 (stage III)
Socinski et al. (19)	3D	CTV 0.5–2 cm; PTV: 1 cm	No	74 Gy	Grade 3 acute esophagitis 16% Grade 3–5 pulmonary toxicity 16%	54%	24.3

*NSCLC: non-small cell lung cancer; ENI: elective nodal irradiation; 2D: two-dimensional; 3D: three-dimensional; IMRT: intensity modulated radiotherapy; Hyperfrx: hyperfractionated; bid: twice daily; RT: radiotherapy; ChemoRT: chemo-radiation; OR: objective response*.

Elective nodal irradiation, which for the most part involves treating areas of mediastinum that do not exhibit tumor as determined by imaging, has been shown to be unnecessary in the era of 3D-CRT. For example, a study by Rosenzweig et al. only identified a 6.1% elective nodal failure among 524 NSCLC patients who underwent radiotherapy to a mean dose of 66 Gy after a median follow up of 41 months (24). In a randomized prospective study by Yuan et al. no statistically significant difference in the rate of elective nodal failure at 5 years following ENI (4%) and involved field irradiation (7%) was observed in stage III NSCLC patients who received concurrent chemo-radiation (25). However, involved field irradiation led to a reduction in radiation pneumonitis (RP) (mainly grade 2–3) from 29 to 17% (*p* = 0.044), and dose escalation from 60–64 to 68–74 Gy. This dose escalation led to statistically significant improvement in local control (55 vs. 38%, *p* = 0.016) and median survival (20.0 vs. 15.0 months, *p* = 0.048). With omission of ENI, dose escalation to 74 Gy with 3D techniques was found to be feasible in RTOG 0117 and CALGB 30105 (18, 19). However, a high rate of severe toxicity was still observed (Table 1). In this regard, the increased toxicity associated with dose escalation was corroborated by findings in RTOG 0617. This may be associated with the limitations of 3D techniques as 3D-CRT without image guidance was allowed in RTOG 0617, which often resulted in sizable gross tumor volume (GTV) to PTV expansion margins (26). Based on these studies, it is proposed that increasing expansion size may also increase the risk of severe treatment related toxicity in the high dose arm of RTOG 0617, which could potentially contribute to the observed decrease in patient survival.

## Strategies to Improve Target Volume Definition through Adaptive Image-Guided IMRT

### Respiratory motion and IMRT

The high incidence of loco-regional failures in NSCLC following radiotherapy may be associated with a high rate of tumor localization error during treatment (27). Such geometric errors may be minimized when respiratory motion is taken into consideration. Lung tumor motion associated with respiration has been well illustrated in multiple studies. In this regard, Seppenwoolde et al. showed that lower lobe tumors, not attached to any rigid thoracic structures, had increased cranial–caudal motion, as compared to upper lobe tumors or tumors attached to rigid structures during treatment (12 ± 6 vs. 2 ± 2 mm, *p* = 0.005) (28). In addition, anterior–posterior tumor motion of >5 mm could be observed in tumors located in the anterior or middle thorax and tumor motion was further complicated by hysteresis. In another study by Liu et al., cranial–caudal motion of >5 mm and ≥1 cm can be observed in 30 and 10% of patients with stage III NSCLC, which is associated with diaphragmatic movement of 1.53 cm on average (29). In a report by the AAPM task group 76, several strategies have been recommended to account for and control respiratory motion, which may be distinct for each individual patient (30).

One commonly used strategy to account for respiratory motion in the treatment of locally advanced NSCLC is 4D CT based treatment planning. Through 4D CT based planning, the range of tumor motion and changes in tumor volume through the entire breathing cycle can be more appropriately and reliably accounted for (29, 31–33). Also, it can potentially decrease the volume of normal tissue included in the PTV and this might be one technique to safely allow for dose escalation to the primary tumor (31). As such, it seems reasonable to suggest that improved accuracy in tumor localization throughout the respiratory cycle could improve tumor control probability (TCP) as “geometric tumor miss” is reduced. In this regard, the maximum intensity projections (MIP) reconstructed from a 4D data set, which reflects the brightest object along each ray on the projection image, may be used for the generation of the internal target volume (ITV) (34) that includes tumor motion into the radiation planning process. However, the outer excursion of respiratory motion may be underestimated by MIP in more advanced tumors and in the setting of irregular breathing patterns by approximately 20% as reported in previous studies (35–37). Thus, the ITV should be generated based on all available 4D CT data.

The utility of 4D CT combined with thoracic IMRT in the treatment of locally advanced NSCLC with chemo-radiation was shown to lower treatment related toxicity and improve patient survival when compared to 3D-CRT in a study from MD Anderson Cancer Center (38). This may be associated with dosimetric advantages of IMRT over 3D-CRT in dose conformity and the sparing of normal organs at risk (OARs) (39–41). As shown by Lievens et al., IMRT can result in significant reduction in the dose to the normal lungs and the spinal cord when compared to 3D-CRT (41). This led to the safe escalation of the prescribed dose (66 Gy in 33 daily fractions) by 8.6–14.2 Gy. Therefore, IMRT may be a strategy for dose escalation in the treatment of locally advanced NSCLC in selected patients through its ability to control radiation dose to critical OARs. This is also suggested by a quality of life (QoL) analysis of RTOG 0617, which demonstrated that the use of IMRT in the setting of dose escalation may improve patients’ QoL, which has been correlated with overall survival (42). Dose escalation with the use of IMRT alone remains to be further investigated in future studies.

### Image guidance with cone-beam CT (CBCT)

Intensity modulated radiotherapy can be used to achieve highly conformal dose distribution with sharp dose gradients and careful daily tumor localization should ensure accurate dose delivery with maximal reduction of treatment margins used for target volume delineation (43–45). This is especially important for dose escalation as radiotherapy often requires 6 weeks or greater to complete in the setting of chemo-radiation for locally advanced NSCLC. In this regard, various image guidance strategies have been proposed and are in clinical use currently (30, 46). Tumor volume, its geometric relation to surrounding OARs, and any anatomical changes during a course of radiotherapy are best imaged with volumetric imaging techniques such as kV or MV cone-beam CT (CBCT). KV CBCT often provides superior soft tissue resolution with compared with MV CBCT due to the prevalence of photoelectric absorption interactions associated with lower beam energies (47). However, MV CBCT may be more helpful in the imaging of regions with high density materials, which produce artifacts in kV CT images (46, 47). As shown by Bissonnette et al., kV CBCT can reduce the set up margin for geometric uncertainties to 3 mm for locally advanced NSCLC patients (48). Such small margin can be safely used under daily image guidance, which reduce set up errors of ≥5 mm from 20–43 to 6% when compared with less-than-daily image guidance (49). Thus, daily image guidance is critical for the safe maximal reduction of set up margins when IGRT, or IG-IMRT is delivered; which will also maximize the possibility of safe dose escalation to the highest dose possible. When compared to other forms of image guidance, kV CBCT was found to be associated with more reliable tumor localization and smaller set up errors (50, 51) and this advantage appears to be most prominent with image registration based on soft tissue in addition to rigid bony registration (51, 52). However, tissue resolution and motion artifacts continue to be a concern for the use of CBCT in the setting of locally advanced NSCLC. These issues may be minimized by 4D CBCT, which remains to be further investigated in the clinical setting (53). One caveat for the clinical adaptation of image-guided IMRT is that there is always a risk for underestimating the range of tumor motion at the time of CT simulation due to the random occurrence of irregular breathing patterns (e.g., random deep inspiration) during the actual delivery of a course of conventionally fractionated radiotherapy. This may potentially lead to the under-dosing of the gross tumor in selected situations of dose painting as very sharp dose gradients are generated at the edge of the tumor targets with intensity modulation. Respiration motion management strategies, such as forced shallow breathing with abdominal compression, may reduce the risk for such geometric misses by limiting diaphragmatic motion to within 5 mm during daily treatment.

### The role of FDG–PET in target volume delineation

FDG–PET/CT has been increasingly incorporated into the treatment planning process to further increase the accuracy of target volume delineation in recent years. FDG–PET imaging is achieved through the detection of a pair of γ rays (511 keV each) that are produced in positron annihilation, which are emitted in 180° to each other (54). FDG–PET is associated with superior sensitivity and specificity (83 and 91%) for tumor detection when compared to CT (64 and 74%) (55). Therefore, PET may provide information for target volume delineation that may be otherwise not available with CT alone.

When compared with CT based treatment planning, PET registered to the planning CT was shown to alter tumor staging in 31% of the patients with stage I–III NSCLC by Bradley et al. (56). In this study, the addition of FDG–PET information resulted in alterations of the treatment volumes in 58% of the patients who were planned for 3D-CRT. Such alterations resulted from the identification of additional regional or parenchymal disease; and the improved tumor definition in the setting of atelectasis, which decreases the unnecessary inclusion of normal lung tissue in the GTV and these findings were corroborated in other studies (57, 58). Also, increased PTV volume, due to the additional nodal disease found with FDG–PET, does not necessarily increase the normal tissue complication probability; while PET data may improve the chance of local control by reducing the likelihood of geometric misses (57). Therefore, the inclusion of FDG–PET information in the treatment planning improves the accuracy of tumor volume delineation, which is critical in the optimization of TCP through IG-IMRT. In addition, PET may lead to nodal GTV reduction and lower doses to the OARs for stage III NSCLC when compared with treatment planning with CT alone, possibly leading to significant iso-toxic dose escalation (59, 60). Based on the evidence illustrated above, FDG–PET inclusion in the treatment planning process may be required in future dose escalation trials for locally advanced NSCLC.

### Adaptive image-guided IMRT

Tumor shrinkage through a course of conventionally fractionated radiotherapy has been well characterized in multiple studies (61–64). In one study, a median GTV reduction of 24.7% after 30 Gy, and 44.3% after 50 Gy was observed in 22 patients with stage I–III NSCLC (78% stage III, 68% squamous cell carcinoma, and 68% underwent concurrent chemo-radiation) (63). In an analysis of 4D CT data collected before and during radiotherapy of at least 6 weeks from patients with stage I–III NSCLC, tumor shrinkage of ≥40% by the end of radiotherapy was observed in 50% of the patients (mainly stage III) (64). Also, increased tumor motion in the cranio-caudal direction increased in half of the patients as tumors shrunk. The observed changes may have an impact on PTV dose coverage and OAR sparing in the treatment of locally advanced NSCLC (62, 65). Therefore, re-planning or ART based on changes observed through daily image guidance is warranted in the treatment of locally advanced NSCLC, which may further maximize safe dose escalation to the gross disease. This may be especially important for IG-IMRT due to the sharp dose gradient generated and small margins for geometric uncertainties used.

Altered fractionation has been previously shown to improve local control and overall survival in the treatment of locally advanced NSCLC (66). This is also supported by the observed clinical outcome following hypo-fractionated radiotherapy for early stage NSCLC (16, 67). Altered fractionation is especially appealing in the setting of dose escalation, as prolonged course of radiotherapy can lead to increased geometric uncertainties, and impairment of local control due to accelerated tumor cell repopulation, which may partially explain the results observed in RTOG 0617 (13, 65). This is also supported by previous RTOG studies on concurrent chemo-radiation, which demonstrated a 2% increase in the risk of death for each day in the prolongation of therapy (68). A dose-per-fraction escalation strategy has been previously proposed to overcome the negative impact of tumor cell repopulation encountered when the total tumor dose is escalated with conventional fractionation. As modeled by Welsh et al., the TCP can be increased to >80% with this strategy in approximately 5 weeks, which would require 100 Gy to be delivered over 10 weeks with conventional fractionation (69).

Early phase and retrospective studies on altered fractionation for radiotherapy alone or combined with chemotherapy in the treatment of stage III NSCLC have shown the feasibility of this strategy (70–78). This is especially true for chemo-radiation delivered with IGRT (74–78). As shown in Table 2, excellent median survival and local control have been frequently observed when hypofractionated, image-guided IMRT was delivered with concurrent chemotherapy. However, severe RP may occur with this approach if low dose irradiation of the normal lungs was not carefully constrained (74, 75). This was shown by Song et al., who observed four RP related deaths following image-guided IMRT delivered with helical tomotherapy (HT) (75). In this study, the rate of ≥grade 3 RP increased from 0 to 35%, when the volume of the contralateral lung receiving 5 Gy (V_5_) was increased to above 60% (*p* = 0.010). This is corroborated in a study by Kim et al., which identified the ipsilateral V_5_, V_10_, V_15_, and the contralateral V_5_ to be significant predictors of RP following HT based IGRT (79). This observation may be partially due to the fan-beam nature of IMRT delivery with HT, which leads to increased low dose irradiation of the normal tissue (80). However, the importance of normal lung sparing from low dose irradiation was observed following linac based IMRT as well, suggesting this to be independent from methods of radiation delivery (81).

**Table 2:** **Chemo-IGRT for locally advanced NSCLC**.

Reference	RT technique	Dose	Clinical outcome	Toxicity
Bral et al. (75)	HT	PTV 70.5 Gy/30 fractions	MS: Stage IIIA vs. III B (21 vs. 12 months, *p* = 0.03). 2-year LPFS: 50%	Late lung toxicity Grade 2 23% Grade 3 16% Two deaths due to grade 5 RP
Song et al. (76)	SIB with HT	GTV: 60–70.4 Gy (2–2.4 Gy/fraction) PTV: 50–64 Gy (1.8–2 Gy/fraction)	Stage III 2 year LC 62% (78% with C-Ch) MS not reached 2 year OS 59% (75% with C-Ch) Only 1 in-field failure was observed	Acute: 14% with grade 3 esophagitis Late: 11% with grade 5 treatment related pneumonitis V_5_ of contralateral lung is a significant predictor of severe RP (*p* = 0.029)
Osti et al. (77)	3D-CRT under kV CBCT image guidance	PTV 60 Gy/20 fractions	Local failure: 37% MS (stage III): 13 months	No patient with >grade 3 treatment related toxicities
Bearz et al. (78)	HT	60 Gy/25 fractions	ORR: 84% MS: 24 months^a^	No RP reported No >grade 3 esophagitis
Donato et al. (79)	HT	67.5–68.4 Gy/30 fractions	Progression in 26% MS 24.1 months (C-Ch)	Acute grade 3 RP 10% Late grade 3 RP 5% No >grade 3 toxicity observed

*^a^ Induction + concurrent chemotherapy; SIB, simultaneously integrated boost; HT, helical tomotherapy; 3D-CRT, 3D conformal radiotherapy; C-Ch, concurrent chemotherapy; LC, local control; OS, overall survival; MS, median survival; LPFS, local progression free survival; RP, radiation pneumonitis*.

One strategy to reduce treatment related toxicities associated with hypo-fractionated radiotherapy may be simultaneously integrated boost (SIB) to the gross tumor through FDG–PET based dose painting and the utility of adaptive IG-IMRT. This is suggested in a dosimetric study of 13 patients with stage III NSCLC (82). In this study, IMRT was shown to reduce the mean lung dose; furthermore, adaptive IMRT with SIB was shown to be superior to 3D-CRT or IMRT alone in dose escalation for larger GTVs, and was able to achieve maximal iso-toxic dose escalation of 17.1 ± 10.1%, which increased TCP by 17.2% on average. This is consistent with image-guided IMRT with the SIB technique that has been previously shown to be feasible by Song et al. (75), and the utilization of adaptive IG-IMRT with SIB in the treatment of locally advanced NSCLC warrants further investigation in future dose escalation trials.

## Novel Use of a Stereotactic Boost and Proton Therapy

Tumor shrinkage after a course of conventionally fractionated radiotherapy may allow for the delivery of a stereotactic boost to the primary tumor in selected patients. In a study by Feddock et al., 10 Gy × 2 fractions, or 6.5 Gy × 3 fractions were delivered following chemo-radiation to a median dose of 59.4 Gy in the treatment of stage II–III NSCLC (83). Although well tolerated in patients with peripheral primary tumors, two deaths due to fatal pulmonary hemorrhage occurred in patients with central lesions. Six local recurrences were observed among 35 patients after a median follow up of 13 months, which corresponds to an actuarial local control of 82.9%. These preliminary results suggest the feasibility of this treatment approach in selected patients, however, patient selection and the most optimal dose fractionation schedule to be used in this setting remains to be further investigated.

Proton therapy has been increasingly investigated in the treatment of lung cancer in recent years due to its advantage in normal tissue sparing over photon therapy (84). In the interaction with tissue, protons enter with lower dose than photon, and deposit the majority of their energy at a certain depth (Bragg peak) with very little exit dose. To be clinically useful, several Bragg peaks can be super-positioned to create a spread-out Bragg peak (SOBP) to cover a specific tumor volume with a desired dose. When compared with photon therapy (3D-CRT or IMRT), proton therapy was shown to significantly reduce the dose to the thoracic OARs, which allowed dose escalation from 63 to 74 Gy within the boundaries of accepted normal tissue dose constraints in stage III NSCLC (85). Intensity-modulated proton therapy (IMPT) may further improve OAR sparing in stage IIIB NSCLC patients, which increases the possibility of dose escalation when compared to IMRT (63–83.5 Gy), and passive scattering proton therapy (PSPT) (74–84.4 Gy) (86). ART may also be indicated in the delivery of PT to improve OAR sparing and target volume coverage (87). In a retrospective study of PT for patients with stage II–III NSCLC, local control of 88.6% was achieved following a median follow up of 16.9 months (88). In this study, a median dose of 78.3 Gy was delivered without any ≥grade 3 toxicity observed. In a phase II study of concurrent chemotherapy and PSPT (74 Gy) for stage III NSCLC, local control of 79.5% and a median overall survival of 29.4 months were observed after a median follow up of 19.7 months (89). In this study, isolated local failure was observed in only 9.1% of the patients, while no grade 4–5 toxicity was observed. While comparable to what is observed in the standard arm of RTOG 0617 in median survival, this may be further improved with adaptive IG-IMPT, which needs to be further investigated in future studies. Due to the unique physical properties of protons, dose distribution in IMPT is very sensitive to tumor motion in relation to the treatment beam scanning motion, which is known as the interplay effect (90). Range uncertainties produced by the interplay effect may lead to under-dosing of the tumor or increased dose to the OARs immediately beyond the range of the proton beam. To account for interplay uncertainties, 4D treatment planning, larger spot size, and fractionated dose schedules have been advocated (91–93). Recently, image guidance for range verification during proton therapy has been shown to be feasible with in-room PET imaging (94). This along with strategies to overcome interplay uncertainties in proton therapy warrants further investigation. However, early clinical experience with proton therapy in the setting of dose escalation for locally advanced NSCLC appears promising.

Although not widely studied, delivering SABR to small peripheral primary tumors and conventionally fractionated radiotherapy combined with concurrent chemotherapy to regional nodal disease in selected cases of locally advanced NSCLC has been endorsed and clinically used by many thoracic Radiation Oncologists. The local control of the primary tumor may be potentially improved as suggested by clinical outcome from SABR for early stage NSCLC (16). At the same time, the regional disease can be addressed with concurrent chemo-radiation. SABR combined with concurrent chemo-radiation, as definitive treatment or a boost to the primary disease, will be suitable for a selected group of patients only. As the mediastinum contains many critical normal structures, which may be at a risk for overdosing along with the lungs if the primary tumor is in their proximity.

## Chemotherapy in the Setting of Dose Escalation

Combining chemotherapy with radiotherapy has been shown to improve local control and patients’ overall survival in previous trials (1, 95). However, chemotherapy, especially taxane-based regimens, has been associated with increased incidence of severe RP (96–99). Severe RP was observed in 47% of the patients treated with concurrent weekly docetaxel and conventionally fractionated radiotherapy to 60–66 Gy delivered with 2D techniques in a phase II study (96). This observation was dose independent, but correlated with the size of initial radiation portals. Consolidation docetaxel was also found to be correlated with increased risk of grade 2–5 RP (14.6 vs. 3.6%, *p* = 0.015) following concurrent chemo-radiation in the retrospective review of the dosimetric data from a randomized prospective study (97). In this study, the mean lung dose was also significantly correlated with the incidence of RP. Furthermore, the increased risk of RP with chemotherapy may be more prominent in patients who are older (98, 99). Therefore, special attention may be necessary to keep the treated volume to as small as possible to minimize the amount of normal lung tissue irradiated to the full dose, which may be especially important in patients who are older than 65 years (99). For the purpose of dose escalation in the setting of chemo-radiation, this may be best accomplished with modern techniques of respiratory motion management and image guidance used in the context of various emerging treatment strategies discussed above.

## Conclusion

Adaptive, image-guided IMRT, when delivered with appropriate respiratory motion management strategies, can effectively reduce the tumor target volume while accurately localize the tumor during a course of chemo-radiation. This allows for dose escalation to the gross disease in the treatment of locally advanced NSCLC as less normal tissue is being irradiated to the prescribed dose. This strategy can be further enhanced with the incorporation of FDG–PET in target volume definition. The utility of stereotactic ablative therapy as a boost or definitive treatment for the primary tumor appears to be feasible in selected patients, while proton therapy, and especially IMPT, appears to be promising and may be superior to photon therapy in dose escalation for the treatment of locally advanced NSCLC. These new treatment approaches remain to be further studied in future clinical trials. Here, we propose the following strategies to be further investigated in selected patients in future trials with respiratory motion management and the incorporation of FDG–PET in the treatment planning required:

(1) Adaptive image-guided IMRT or IMPT delivered in a simultaneously integrated fashion with concurrent chemotherapy.
(2) Stereotactic boost to the primary tumor to be delivered prior or after a standard course of concurrent chemo-radiation to 60 Gy, or given in between a split course of concurrent chemo-radiation that is hypofractionated, image-guided, and intensity modulated.
(3) Stereotactic ablative radiotherapy to the primary tumor to be followed by concurrent chemotherapy and image-guided IMRT to the regional nodal disease.

## Conflict of Interest Statement

The authors declare that the research was conducted in the absence of any commercial or financial relationships that could be construed as a potential conflict of interest.
